# Assessment of Salivary Levels of RANKL and OPG in Aggressive versus Chronic Periodontitis

**DOI:** 10.1155/2019/6195258

**Published:** 2019-04-28

**Authors:** Alexandra Cornelia Teodorescu, Ioana Martu, Silvia Teslaru, Diana Cristala Kappenberg-Nitescu, Ancuta Goriuc, Ionut Luchian, Maria Alexandra Martu, Sorina Mihaela Solomon, Silvia Mârțu

**Affiliations:** Grigore T. Popa University of Medicine and Pharmacy Iasi, Faculty of Dental Medicine, 16 Universității Str., 700115, Romania

## Abstract

RANKL (receptor activator of nuclear factor kappa-*β* ligand) and OPG (osteoprotegerin) are two proteins involved in bone remodelling. During the active phase of periodontal disease, an imbalance between the ratios of the two elements can be noticed. While the expression of RANKL is elevated compared with that of OPG, the RANKL is available to bond with RANK (receptor activator of nuclear factor kappa-*β*). This study was conducted on 41 patients: 19 with generalized aggressive periodontitis, 18 with severe chronic periodontitis, and 4 periodontal healthy subjects. For each patient included, we determined the salivary levels of RANKL and OPG with the help of two Human ELISA kits. The results show that the patients affected by periodontitis, either aggressive or chronic, have significant higher values of RANKL and RANKL/OPG ratio. This values correlate with the local inflammation status.

## 1. Introduction

All bones of the human skeleton, including the maxillary bones, undergo a constant and continuous remodelling process throughout the life, so that they can adapt to the biomechanical forces that are generated. Bone remodelling consists in destroying the old bone that has suffered micro lesions and replacing it with a new one, more resistant from a mechanical perspective.

Generally speaking, bones are made by two types of tissues: the compact bone, exterior part, dense, and tough and the trabecular bone, spongy and less dense than the cortical. All bones consist of “support” cells (osteoblasts or osteoclasts), “remodelling” cells (osteoclasts), a collagen matrix, and noncollagenic proteins called “osteoid,” plus inorganic mineral salts inside the matrix. Bone remodelling is a process that must take place in an organized manner which depends directly on the growing and activation of osteoclasts and osteoblasts [1].

RANK, a cell surface receptor, makes the precursor osteoclastic cells to develop into completely differentiated osteoclasts when it is activated by its ligand (RANKL). This ligand is part of the TNF (tumor necrosis factor) superfamily and it is vital to the formation of osteoclasts. It is a key molecule that favours the interaction between osteoblasts and osteoclasts in bone remodelling [1].

RANKL is a 314 amino acid polypeptide, coded by the TNKSF11 gene and expressed in a membrane protein that is also part of the TNF family [2, 3].

Osteoprotegerin (OPG) is another protein produced and released by the osteoblasts, which acts like a “false target” to prevent RANK from getting in contact with RANKL. OPG plays a vital role in inhibiting the differentiation and activation of osteoclasts and, by that, in bone resorption. OPG is a dimeric glycoprotein from the TNF family that, by preventing the bonding of RANK to RANKL, inhibits the recruiting, proliferation, and activation of osteoclasts.

The RANKL/RANK/OPG system is one of the most important discoveries of the last decade in bone biology [4]. When there are imbalances in the optimal RANKL/OPG ratio, bone resorption is augmented as it happens in case of postmenopausal osteoporosis, Paget disease, bone cancerous metastasis, and rheumatoid arthritis [1, 5–7].

Periodontitis is an inflammatory disease that affects the surrounding and supporting tissues of the teeth. Some bacteria are well known to be periodontal pathogens: *Aggregatibacter actinomycetemcomitans*, *Porphyromonas gingivalis*, *Tannerella forsythia*, *Treponema denticola*, and *Prevotella intermedia*. Depending on the type of periodontitis, aggressive or chronic, these bacteria can be found in different associations, but always above the pathogenic thresholds [8, 9]. The bone lysis is highly correlated with the periodontal inflammation and can be influenced by the interactions between pathogenic bacteria, the immune host response, and other infections. During periodontal disease, the evolution of the inflammatory immune response implies a wide range of inflammatory and immunologic mediators, such as nitric oxide, cytokines, metalloproteinases, and chemokines [10–19].

The progression of inflammation can influence the formation and activation of osteoclasts, the key cells in bone resorption. This process is influenced by the expression of RANK, found in osteoclasts, dendritic cells, fibroblasts, and T lymphocytes, by the augmentation of RANKL in osteoblasts and T lymphocytes and by the decreased expression of the soluble receptor OPG found in osteoblasts and endothelial immune cells [3]. It was also shown that the presence of important periodontal pathogens such as *Porphyromonas gingivalis* and *Prevotella intermedia* increases the RANKL expression in human periodontal ligament cells [20] and that RANKL levels increase with inflammation and periodontal degradation in the presence of *Aggregatibacter actinomycetemcomitans* [21].

The aim of this study was to evaluate the changes in the two salivary proteins, RANKL and OPG, during generalized aggressive periodontitis (GAP) and severe chronic periodontitis (SCP), and to explore their correlation with the bleeding on the probing index (BOP). The two biomarkers are directly involved in bone remodelling and have an antagonist action.

## 2. Materials and Methods

This study was conducted on 41 patients, with ages between 20 and 50 years old. The inclusion of patients in this study was made by 3 criteria: the age between 18 and 50 years, a periodontal diagnosis of GAP or SCP, and 20 or more teeth present. In this study, we added 4 more patients with the same age and tooth criteria, but without any form of periodontitis (healthy patients). Because we considered these patients as a control group, they could have presented the worst localised form of gingivitis. This diagnosis meant a bleeding of less than 30% of probing sites.

The exclusion criteria were as follows:

- Periodontal treatment in the last 6 months
- Systemic antibiotics in the last 3 months
- Pregnant or breastfeeding women
- Important systemic diseases such as leukaemia, malign tumors, and recent acute cardiac pathology (in the last 6 months)
- Anticoagulant therapy
- Ongoing bisphosphonate therapy or in the last 12 months

The steps of this study were explained to all the patients included, and all of them read and signed an informed consent.

After making the periodontal diagnosis, the patients were divided into 3 groups: GAP, 19 patients; SCP, 18 patients; and H (healthy), 4 patients. For each patient, we determined the BOP index. This index was made with the use of a WHO probe, on the Ramfjord teeth (1.6, 1.1, 2.6, 3.6, 3.1, and 4.6) by probing on every lateral aspect of each tooth. The sum of all surfaces with bleeding on probing was divided by 24 (the total number of probed surfaces), using the following formula:

(1) $$\begin{array}{c} \text{BOP}=\frac{\text{number of surfaces with bleeding}}{24}*100. \end{array}$$

From each patient enrolled in this study, we collected unstimulated saliva. The saliva collection was done in the morning, after an all-night fast. The patients could have drunk water, but no later than one hour before the saliva collection. The saliva that we collected was unstimulated; the patients were instructed to gather saliva on the floor of their mouth and then spit it into a sterile container a few times until enough saliva was collected.

The saliva samples were processed in a laboratory respecting the manufacturer's indications. For their analysis, we used the Human RANKL ELISA kit and Human OPG ELISA kit from Elabscience.

The study protocol was approved by the Research Ethics Committee at “Grigore T. Popa” University of Medicine and Pharmacy in Iasi, Romania, with registration number 5328/8.03.2018.

## 3. Results and Discussion

**Figure 1:**
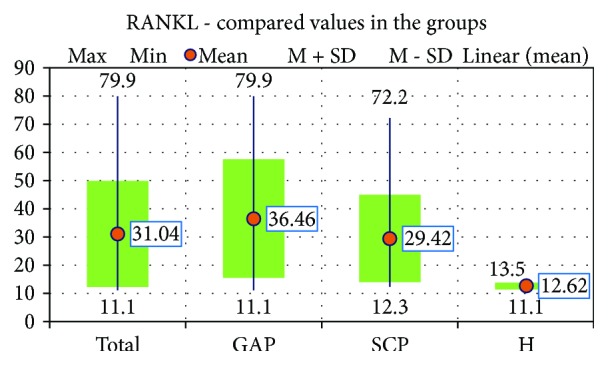
The values of RANKL in the 3 study groups.

First of all, we analysed the salivary RANKL levels in the 3 study groups. Clearly, the highest value was noticed in the GAP group, with a mean of 36, 46 ± 20,924 pg/ml, and the lowest value was registered in the H group, with a mean of 12, 62 ± 1,068 pg/ml (Figure 1). The mean values were significantly higher in the GAP (*p* < 0,001) and SCP (*p* = 0,001) groups, compared with the H group, but we found no significant differences between the two periodontitis groups.

**Figure 2:**
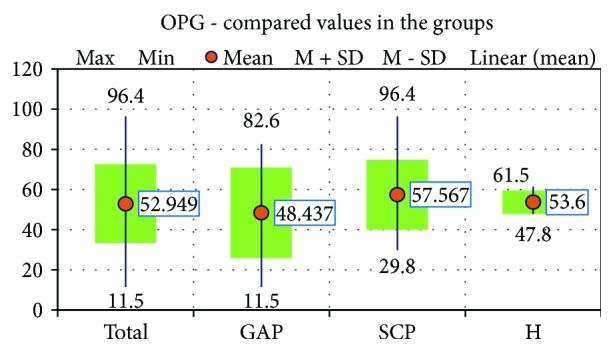
The compared OPG values in the 3 study groups.

The next step was to compare the salivary means of OPG in the 3 study groups, and we found no significant differences of the mean values of this salivary protein between the 3 groups, as it is shown in Figure 2.

**Figure 3:**
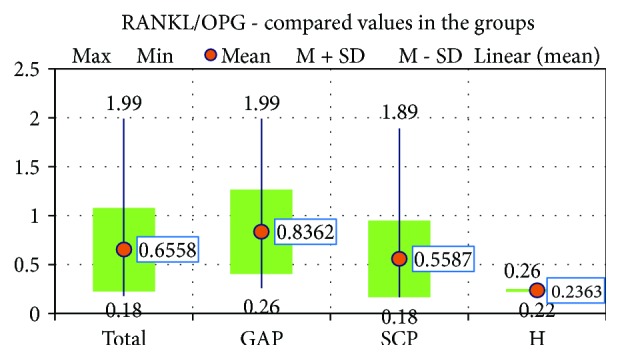
The compared values of the RANKL/OPG ratio in the 3 study groups.

We also looked into the RANKL/OPG ratio in all 3 study groups. It is visibly clear that the highest mean value was for the GAP group (0, 8362 ± 0, 43072 pg/ml), significantly higher than the SCP group (*p* = 0,039) and the H group (*p* = 0,009) (Figure 3).

**Figure 4:**
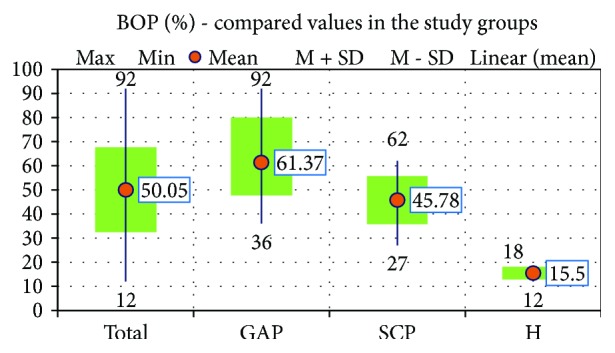
The mean values of the BOP for the 3 study groups.

For the patients enrolled in this study, we also determined the BOP index, which we then compared between the 3 study groups. Its mean value was highest in the GAP group (61, 37 ± 13,736) and lowest in the H group (15, 5 ± 2,517) (Figure 4). The BOP index was significantly higher in the two periodontitis groups when compared to the H group (*p* < 0,001), and it was also significantly higher in the GAP group compared to the SCP group (*p* < 0,001).

**Figure 5:**
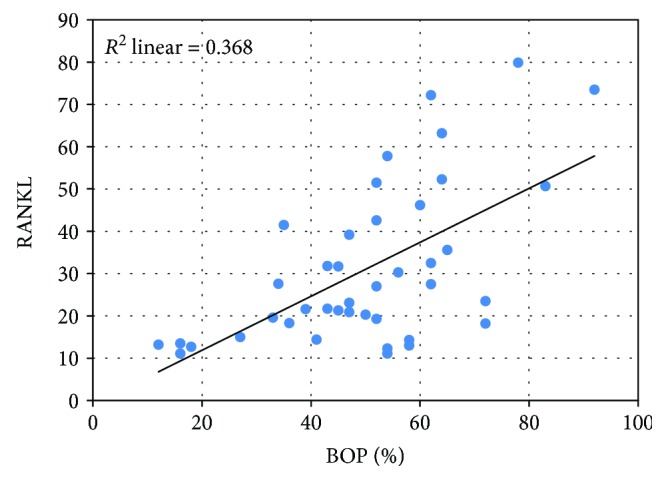
The strong, directly proportional correlation between RANKL and BOP.

**Figure 6:**
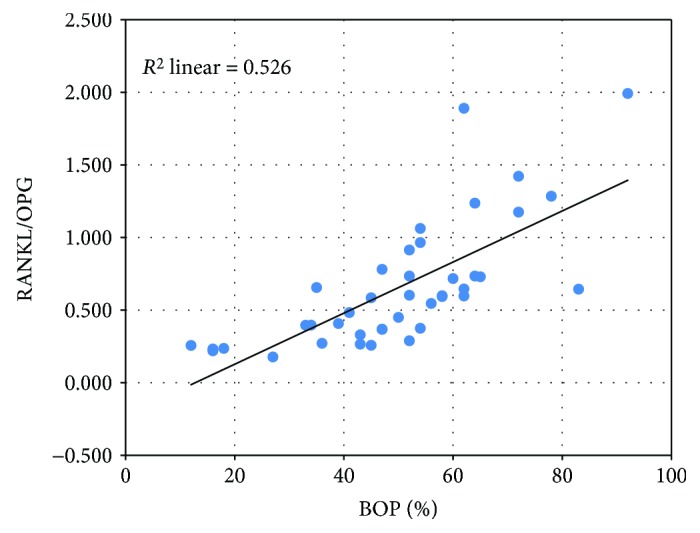
The strong, directly proportional correlation between the RANKL/OPG ratio and BOP.

**Figure 7:**
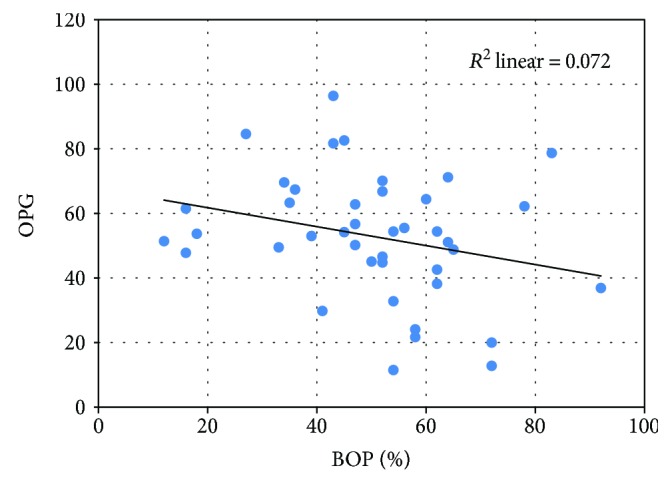
The weak, inversely proportional correlation between OPG and BOP.

In order to determine whether there was a correlation between BOP, RANKL, OPG, and their ratio, we build a linear regression model and we calculated the correlation coefficient Pearson *r*. A very strong, directly proportional, and statistically significant correlation was noted between BOP and RANKL (*p* < 0,001) (Figure 5) and BOP and RANKL/OPG ratio (*p* < 0,001) (Figure 6) and a weak, inversely proportional, and nonsignificant correlation between BOP and OPG (Figure 7).

Periodontitis is one of the most frequent oral diseases. It is a very important health issue that affects the patients' quality of life [22]. Periodontitis is diagnosed by clinical and radiographic criteria, which are not sufficient in determining the activity of the disease and the patient's susceptibility to the disease progression. The biomarkers in the oral fluids have the potential to offer more information than the standard clinical indexes [23].

All studies available now have found that RANKL and OPG can be easily detected in the gingival tissues and the biological fluids (saliva, crevicular fluid, and serum). A high RANKL/OPG ratio is a sign of periodontitis. This ratio is not suddenly and greatly modified after a successful periodontal therapy, but it can serve as a tool to highlight the presence of an untreated periodontitis or a history of one. The increase of this ratio can mean that the molecular mechanisms of bone resorption are still activated and that the affected sites still present a risk of relapse [24].

All tissues affected by periodontitis show higher values of RANKL and lower values of OPG compared to healthy one. The RANKL/OPG ratio was found to have a 2.2-fold increase in the patients with chronic periodontitis, compared to healthy subjects [25]. In the present study, this ratio had significantly higher values in the GAP group compared to the SCP or H groups. These findings are correlated with other studies which found that the RANKL/OPG ratio had higher values for patients with aggressive and chronic periodontitis, when compared to healthy subjects or those affected only by gingivitis [26]. Unfortunately, most recent studies had their focus on comparing patients with chronic periodontitis and healthy subjects and not aggressive periodontitis versus chronic periodontitis versus healthy subjects, as we have done in our study.

Gabr et al. in 2017 have found a significant increase of the RANKL protein in the saliva of SCP patients, compared with healthy ones (*p* < 0,001) and an increase that is not surprising considering that this protein plays a key role in the tissue degradation happening in periodontitis [27].

In 2016, İlarslan et al. found that RANKL and the RANKL/OPG ratio had greater values in the periodontitis group (*p* < 0,001) when compared to the healthy subjects, but found no significant differences concerning the values of OPG [28]. Other studies have shown high levels of RANKL for periodontitis patients, but no differences for OPG [25, 29], as in our study.

In 2013, Tabari et al. [22] show results similar to the current study: a significant increase of RANKL and RANKL/OPG ratio and a nonsignificant increase of OPG, but the salivary values of these biomarkers they reported are very different: 266 ± 48 pg/ml RANKL in the chronic periodontitis group, 207 ± 83 pg/ml RANKL for the healthy subjects, 2, 1 ± 1.0 pg/ml OPG in the CP group, and 2, 20 ± 7, 78 pg/ml OPG in the H group. Similar values as those of our study were reported by Hassan et al. in 2015 [30], OPG 64, 5 ± 20, 9 pg/ml for the CP patients, and by Al-Ghurabi and Mohssen [31], in the same year, with a mean value for RANKL of 56, 8 pg/ml in the CP group.

Other important studies have looked into the level of RANKL and OPG in the GCF (gingival crevicular fluid). In 2007, Bostancı et al. [26] compared GCF samples of individuals with healthy periodontium, gingivitis, aggressive periodontitis, and chronic periodontitis by means of ELISA and found that RANKL levels and the RANKL/OPG ratios were lower in the healthy and gingivitis group and increased in all forms of periodontitis. More recently, Gabr et al. [27] found that moderate and severe periodontitis patients have higher levels of salivary and GCF RANKL (and also a higher RANKL/OPG ratio) than healthy individuals. Together with Al-Ghurabi and Mohssen [31] and Mogi et al. [32], all these studies back up our finding that periodontitis, be it aggressive or chronic, is correlated with increased levels of RANKL protein implicated in bone destruction.

Opposite to our findings that RANKL levels and the RANKL/OPG ratio are positively correlated with bleeding on probing, Bostancı et al. found in 2011 an inversely proportional relation between the crevicular levels of RANKL, the RANKL/OPG ratio, and PBI (papillary bleeding index) and reported that the beneficial results of the periodontal therapy were not correlated with a decrease in the RANKL/OPG ratio [33]. In 2004, Mogi et al. found no correlation between GCF RANKL levels and gingival inflammation [32].

A decrease in the RANKL/OPG ratio could provide new therapeutic approaches in the field of periodontology and establish new protocols. In a study made by Chatterjee et al. in 2014, it has been shown that fish oil, full of omega 3 fatty acids, may determine a decrease of this ratio due to its anti-inflammatory effect, bettering the periodontal status [34]. The possibility of using a modulation therapy which could determine the decrease of RANKL levels and the increase of OPG levels should open, in the following years, new perspectives in the periodontal approach of the patient.

## 4. Conclusions

The RANK/RANKL/OPG system proved to play a major role in the production and activation of osteoclasts, which are the key elements in charge of regulating the bone resorption.

An increase in the RANKL/OPG ratio, although associated with the presence of periodontal disease, was not able to distinguish between the mild, moderate and severe forms of periodontitis but can still be used as an additional diagnostic tool for detecting the disease.

## Data Availability

The data used to support the findings of this study are available from the corresponding author upon request.
